# Social Support and Suicide Risk Among Chinese University Students: A Mental Health Perspective

**DOI:** 10.3389/fpubh.2021.566993

**Published:** 2021-02-17

**Authors:** Haiyun Chu, Yanjie Yang, Jiawei Zhou, Wenbo Wang, Xiaohui Qiu, Xiuxian Yang, Zhengxue Qiao, Xuejia Song, Erying Zhao

**Affiliations:** Medical Psychology Department of the Public Health Institute of Harbin Medical University, Harbin, China

**Keywords:** suicide risk, social support, anxiety, depressive symptoms, university students

## Abstract

**Background:** World Health Organization recognizes suicide as a public health priority. This study aimed to investigate the risk life events which led university students to consider suicide and explore the protective mechanism of social support (including subjective support, objective support, and support utilization) on suicide risk.

**Methods:** Three thousand nine hundred and seventy-two university students were recruited in Harbin, China. Social Support Rating Scale, Self-Rating Anxiety Scale, the Beck Depression Inventory, and the 25-item scale of suicide acceptability were used to collect participants' information. Descriptive statistics, Pearson's correlation, and mediation analysis were employed for statistical analysis.

**Results:** “Drug addict,” “infected with HIV,” and “incurable illness” were the top three events that led university students to consider suicide. Social support played an important protective role against suicide risk. Subjective support and support utilization had total effects on suicide acceptability, including direct and indirect effects. Anxiety (indirect effect = −0.022, 95% CI = −0.037 ~ −0.009) and depressive symptoms (indirect effect = −0.197, 95% CI = −0.228 ~ −0.163) mediated the relationship between subjective support and suicide acceptability; meanwhile, the association between support utilization and suicide acceptability was mediated by anxiety (indirect effect = −0.054, 95% CI = −0.088 ~ −0.024) and depressive symptoms (indirect effect = −0.486, 95% CI = −0.558 ~ −0.422). However, the protective impact of objective support worked totally through decreasing anxiety (indirect effect = −0.018, 95% CI = −0.035 ~ −0.006) and depressive symptoms (indirect effect = −0.196, 95% CI = −0.246 ~ −0.143). Moreover, the mediation effects of depressive symptoms had stronger power than anxiety in the impact of social support on suicide risk.

**Conclusions:** Among Chinese university students, suicide acceptability was elevated when there was a health scare. Social support effectively reduced suicide risk *via* decreasing anxiety and depressive symptoms. From the mental health perspective, families, peers, teachers, and communities should work together to establish a better social support system for university students, if necessary, help them to seek professional psychological services.

## Introduction

According to the latest available data from the World Health Organization (WHO), there are close to 800,000 suicide deaths every year, numbering one person every 40 s (1). Suicide has continued to be a serious public health problem around the globe, which is the second leading cause of death among 15–29-year-olds worldwide. In China, suicide has become the first cause of death in the Chinese population aged 15–34 (2). Adolescence and young adulthood are a time for development and changes in biological, psycho-social, and spiritual aspects of life. University students are in the most critical onset period for various mental health problems and experience high suicide risk (3) because of a new academic environment, leaving home, new relationships, and academic pressure (4, 5). Suicide risk has been the focus of increasing attention in research and public awareness campaigns over the past decade (6). Prevention of suicide is significantly necessary. WHO reported that many suicides happened impulsively in moments of crisis with an inability to deal with life stresses, such as chronic pain, illness, financial problems, and relationship break-up, etc. (1) As a reliable screening tool for early suicide risk (7), suicide acceptability refers to the possibility of accepting suicide in various stressful life events (8). To assess the suicide acceptability among university students, Phillips summed up 25 events in which they would consider suicide: HIV/AIDS, incurable illness, drug dependence, a burden on others and no future hope, chronic mental illness, elderly and no family to provide support, severe depression, large debt due to gambling, raped, imprisoned, homosexuality, death of a spouse, frequently beaten by the spouse, severe economic loss, poor with no prospects for improvement, chronic alcohol abuse, changed with a crime, marriage plans interfered with by others, severe loss of face, major fright, serious interpersonal conflict, divorce, penalized at work, the suicide of family members or friends and failed university entrance exam (8). Given the insidious suicidal behavior, suicide acceptability can widely and accurately identify high-risk populations before individual encounters real stressful events (9).

WHO made suicide prevention a high priority on the global public health agenda (1), and social support was recognized as a significant protective factor of suicide prevention (10). Social support is defined as the care, support, and assistance from families, friends, and communities (11). Adolescence is stressful for many young people and involves identity formation and changes in sexuality, self-concept, social roles, and relationships (12, 13). Social support, including objective support and subjective support, may strengthen adolescents to process their stress, facilitate coping, and increase adjustment (14). Research on suicide found that: most university students with a history of suicide attempts suffered from a poor social support system and had a positive attitude toward suicide behavior; while receiving more social support, the risk of suicide would be reduced (15). Gauvin suggested that tangible assistance was the strongest predictor for suicide prevention (16). Of course, no matter how excellent the social support network is, it would not be very worthy until it is utilized. A close network of families, friends, and community connections is highly valued in Chinses society, which is important for adolescents and young adults to resist and effectively cope with the stressful events related to mental health (17).

Despite growing evidence for the positive impact of social support among adolescents and young adults, the underlying mechanisms remain poorly defined. The interpersonal theory of suicide (18), an explanation from the relationship perspective, emphasizes the positive effects of relationships, and the negative effects of mental health problems.

Outbreaks of major life events put a lot of pressure on an individual, leading to many psychological problems and challenges (19). Anxiety and depressive symptoms are the most common mental problems of adolescents and young adults (20), which are generally caused by negative life events and a lack of social support (21). Anxiety symptoms are an adaptive emotional response to physical or mental challenges and have been shown to have protective functions and damaging effects, producing anxiety disorder (22). Depressive symptoms are about bad mood and negative beliefs and may lead to some consequences, such as pessimism, personal quarrel, and even suicide behavior (23, 24). Studies have reported increasing rates of mental disorders in the university setting. For instance, Wahed et al. revealed that the prevalence of anxiety and depression in medical Fayoum University students were 64.3% and 60.8%, respectively (25). A lot of behavior problems—such as drop-out of school, internet addiction, narcotics, alcohol, and other substance abuse—occur because of anxiety, depression, and other mental health issues (26, 27). Suicide behavior is the most severe consequence of mental disorders. Evidence supported that mental health services could effectively reduce suicide rates (28), such as pharmacotherapy, cognitive therapy, and behavior therapy (29, 30). A review summarized the role of psychopharmacons in suicide prevention and showed 40–81% reductions for suicide risk after taking antidepressants, and psycho-social interventions further improved the effect (31). Mental health disparities are present early in adolescence and increase throughout young adulthood. Early intervention of these mental problems must be a priority.

According to the interpersonal theory of suicide, high-quality interpersonal connections benefit individuals' mental health (18). An excellent social support system could protect adolescents and young adults under interpersonal life stress and psychological distress (32, 33). With the combined action of subjective support and objective support from families, friends, classmates, teachers, and communities, university students will get healthier in physiology and psychology. The present study aimed to investigate the risk life events which led university students to consider suicide and explore the protective mechanism of social support (including subjective support, objective support, and support utilization) on suicide risk. The hypothesis was: anxiety and depressive symptoms would play mediation roles in the association between social support and suicide acceptability in Chinese university students.

## Materials and Methods

### Participants and Processes

This study was cross-sectional research and conducted in Harbin, Heilongjiang Province, China. We recruited the participants by stratified random cluster sampling method and done as follows: First, six universities were selected randomly from a dozen universities; Next, we calculated the distribution of samples from these universities as the proportion of participants; Finally, we selected classes from all grades randomly, and students in these classes were recruited as participants of this study. Identified students were notified by administrators to come to the classroom at a specific time. The students were told about the purposes, content, and possible effects of this study. The survey was completely voluntary, and students had the right to refuse to participate in the survey. All participants were advised that the questionnaires should be finished within 30 min. Once completed, the survey was briefly reviewed by coordinators. Eventually, we recruited 4,000 university students for our study.

### Ethics Statement

The research met the ethical guidelines, and its approval was granted by the Ethics Committee of Harbin Medical University. All participants have signed informed consent forms and been told about the research's purposes, meanings, and content.

### Instruments

The social support level was assessed with the Social Support Rating Scale, which was created by Xiao (34). It included three dimensions (subjective support, objective support, and support utilization). There were ten items. And seven items were answered on a 4-point Likert scale, and the others were answered in a special way (calculating numbers of support sources). Responses were calculated, and the scores of subjective support, objective support, and support utilization ranged 8–32, 1–22, and 3–12, respectively (35). Higher scores indicated a higher social support level.

Self-Rating Anxiety Scale (36), created by Zung, was used to measure the anxiety symptoms of university students. There were 20 items, which was evaluated by 1–4 score. In order to generate the index score, the raw score was multiplied by 1.25, and only the integer part was kept. The cut-off score for anxiety symptoms was an index score of 50 (original raw score of 40) (37). Higher index scores on the SAS indicated increasing levels of anxiety.

The Beck Depression Inventory (38), created by Aaron T. Beck, was used to assess the depressive symptoms of university students in this study. There were 21 items, which were evaluated by a 0–3 score. The total score ranged from 0 to 63, and the cut-off score for depression was 14 (39). Higher scores indicated a higher depression level.

Suicide acceptability level was identified with the 25-item scale of suicide acceptability (8). This scale was developed by the Beijing Suicide Research and Prevention Center and was used to assess the likelihood that students would consider suicide when they encountered different types of life problems. All items were answered on a 5-point Likert scale. The score of scale was obtained by expressing the raw score converted to 100 points scale. Higher scores indicated a higher suicide acceptability level.

### Data Analysis

All data analyses were performed by SPSS 24.0 and MPLUS 7.0. The results were evaluated at a significance level of *P* < 0.05 (two-tailed).

Descriptive statistics were used to describe the demographic characteristics (age, gender, major, family economic situation, religion, and love status), social support, anxiety, depressive symptoms, and suicide acceptability of this study samples. Pearson's correlation was performed for assessing associations between social support, anxiety, depressive symptoms, and suicide acceptability. Parallel multiple mediation analysis was employed to test the hypothetical model. Based on 1,000 bootstrap samples, direct and indirect effects were evaluated with 95% bias-corrected confidence intervals. When the 95% confidence interval (CI) did not contain zero, the mediation effect was significant.

## Results

### Sample Characteristics

Three thousand nine hundred and seventy-two valid questionnaires were collected from 4,000 (effective response rate: 99.3%). There were 1,929 (48.6%) males and 2,043 (51.4%) females in this study. The average age of university students was 20.71 ± 1.397 years, ranging from 17 to 23. The 3,972 university students were from different majors as follows: Science 626 (15.8%), Engineering 1,690 (42.5%), Agronomy 463 (11.7%), Medicine 306 (7.7%), Literature 548 (13.8%), and Management 339 (8.5%). There were 337 (8.5%) religious and 3,635 (91.5%) non-religious in this study. Of these students, 1,825 (45.9%) were not in love, 2,136 (53.8%) were in love, and 11 (0.3%) are in other situations. In addition, 283 (7.2%), 2,687 (67.6%), and 1,002 (25.2%) students reported that they were in good, moderate, and poor family economic situations, respectively.

As Table 1 showed, according to the mean score of each item, all university students considered “drug addict,” “infected with HIV,” and “incurable illness” as the three situations that would most likely lead them to consider suicide. Table 2 indicated that the mean score of suicide acceptability was 34.83 ± 10.05, ranging from 1 to 100. University students of this study showed a moderate possibility of accepting suicide in various stressful life events. The mean scores for anxiety symptoms and depressive symptoms were 39.98 ± 5.74 and 27.19 ± 5.84. According to the norms for scales, university students had a low level of anxiety symptoms and a high level of depressive symptoms. Moreover, the mean scores for subjective support, objective support, and support utilization were 18.79 ± 3.39, 6.57 ± 1.63, and 8.11 ± 1.85, respectively. Chinese university students presented better subjective support and support utilization and a low objective support level in this study.

**Table 1:** Suicide acceptability of Chinese university students showing the likelihood that they would consider suicide if they experienced different life circumstances (*n* = 3,972).

**Event**	**Rank**	**Mean**	***SD***
Drug addict	1	2.22	1.377
Infected with HIV	2	2.16	1.296
Incurable illness	3	1.83	1.156
Raped	4	1.67	1.041
Serious depression	5	1.58	0.884
Chronic mental illness	6	1.56	0.899
Spouse died	7	1.46	0.763
Major gambling loss	8	1.45	0.847
Burden to others that will not change	9	1.44	0.778
Old but not one to care for you	10	1.41	0.844
Long-term over drinking	11	1.39	0.775
Homosexuality	12	1.37	0.907
Imprisoned	13	1.36	0.736
Often beaten by spouse	14	1.28	0.682
Major financial loss	15	1.25	0.561
Friend suicides	16	1.23	0.562
Major conflict with other	17	1.22	0.522
Poor with no hope of improvement	18	1.20	0.538
Accused of crime	19	1.18	0.494
Divorce	20	1.13	0.402
Major fright	21	1.12	0.390
Failed university entrance	22	1.10	0.371
Marriage plans interfered with by others	23	1.08	0.328
Major loss of face	24	1.08	0.308
Penalized at work	25	1.06	0.250

**Table 2:** Correlations of social support, anxiety, depressive symptoms, and suicide acceptability among Chinese university students (*n* = 3,972).

	**1**	**2**	**3**	**4**	**5**	**6**
1. Subjective support	1					
2. Objective support	0.140^a^	1				
3. Support utilization	0.281^a^	0.152^a^	1			
4. Anxiety symptoms	−0.137^a^	−0.049^a^	−0.178^a^	1		
5. Depressive symptoms	−0.225^a^	−0.095^a^	−0.303^a^	0.531^a^	1	
6. Suicide acceptability	−0.263^a^	−0.055^a^	−0.236^a^	0.237^a^	0.367^a^	1
Mean	18.79	6.57	8.11	39.98	27.19	34.83
SD	3.39	1.63	1.85	5.74	5.84	10.05

a *P < 0.01*.

### Pearson's Correlation Analysis of Social Support, Anxiety, Depressive Symptoms, and Suicide Acceptability

Correlations of the study variables were displayed in Table 2. Results showed that correlations between social support (subjective support, objective support, and support utilization) and suicide acceptability were significantly negative (*P* < 0.01). But the relationship between objective support and suicide acceptability was in the small association size (*r* = −0.055, *P* < 0.01). In addition, anxiety and depressive symptoms were associated with suicide acceptability (*P* < 0.01), as well as social support among university students (*P* < 0.01).

### Parallel Multiple Mediation Analysis

**Figure 1:**
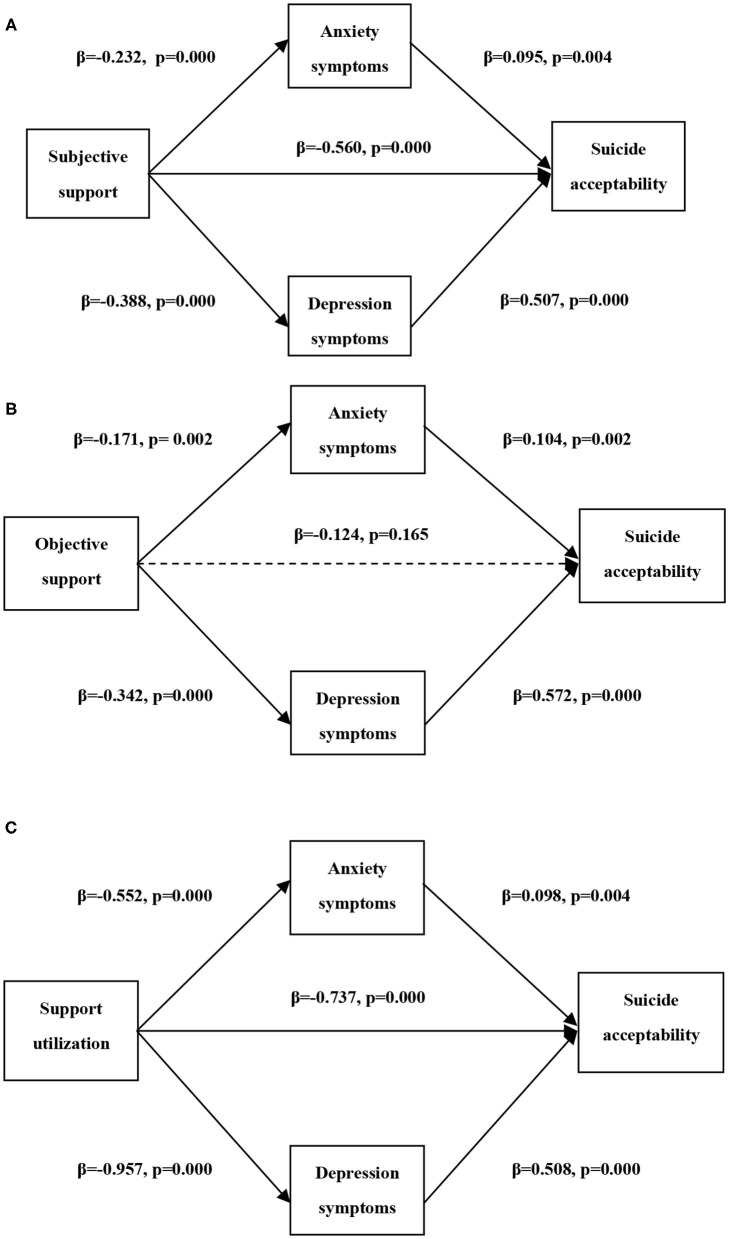
The parallel multiple mediation models of the relationship between social support and suicide acceptability among Chinese university students. **(A)** the mediation model of the relationship between subjective support and suicide acceptability, **(B)** the mediation model of the relationship between objective support and suicide acceptability, and **(C)** the mediation model of the relationship between support utilization and suicide acceptability. All models were built after controlling age, gender, love status, major, family economic situation, and religion.

Figure 1 showed the final parallel multiple mediation model of the relationship between social support and suicide acceptability among Chinese university students, with age, gender, major, family economic situation, religion, and love status controlled. As Figures 1A,C displayed, subjective support and support utilization not only had a significant direct negative effect on suicide acceptability but also had indirect effects *via* anxiety and depressive symptoms. While as Figure 1B presented, the protective impact of objective support on suicide acceptability totally worked through anxiety and depressive symptoms. The statistical goodness of fit of the multiple mediation model was as follows: (1) in the mediation model of subjective support on suicide acceptability, χ^2^/df =7.362, CFI = 0.968, TLI = 0.935, RMSEA = 0.040, and SRMR = 0.032; (2) in the mediation model of objective support on suicide acceptability, χ^2^/df =7.577, CFI = 0.961, TLI = 0.922, RMSEA = 0.041, and SRMR = 0.034; and (3) in the mediation model of support utilization on suicide acceptability, χ^2^/df =7.656, CFI = 0.968, TLI = 0.936, RMSEA = 0.041, and SRMR = 0.032.

Additionally, mediation effects of anxiety and depressive symptoms were showed in Table 3. Anxiety (indirect effect = −0.022, 95% CI = −0.037 ~ −0.009) and depressive symptoms (indirect effect = −0.197, 95% CI = −0.228 ~ −0.163) mediated the relationship between subjective support and suicide acceptability; meanwhile, the association between support utilization and suicide acceptability was mediated by anxiety (indirect effect = −0.054, 95% CI = −0.088 ~ −0.024) and depressive symptoms (indirect effect = −0.486, 95% CI = −0.558 ~ −0.422). However, the protective impact of objective support worked totally through decreasing anxiety (indirect effect = −0.018, 95% CI = −0.035 ~ −0.006) and depressive symptoms (indirect effect = −0.196, 95% CI = −0.246 ~ −0.143). Moreover, the mediation effects of depressive symptoms had a stronger power than anxiety in the protective impact of all kinds of social support on suicide acceptability among university students. In the effects of subjective support, objective support, and support utilization on suicide acceptability, the mediation effects of depressive symptoms accounted for 25.3%, 58.2%, and 38.1% of total effects, respectively.

**Table 3:** Indirect effects of anxiety and depressive symptoms in the associations between social support and suicide acceptability (*n* = 3,972).

	**Effect**	**SE**	***P***	**95% bootstrap CI**
**Subjective support**				
Total effect	−0.779	0.047	0.000	(−0.851, −0.698)
Indirect effect of anxiety symptoms	−0.022	0.008	0.008	(−0.037, −0.009)
Indirect effect of depressive symptoms	−0.197	0.020	0.000	(−0.228, −0.163)
**Objective support**				
Total effect	−0.337	0.092	0.000	(−0.486, −0.183)
Indirect effect of anxiety symptoms	−0.018	0.009	0.044	(−0.035, −0.006)
Indirect effect of depressive symptoms	−0.196	0.033	0.000	(−0.246, −0.143)
**Support utilization**				
Total effect	−1.277	0.081	0.005	(−1.406, −1.137)
Indirect effect of anxiety symptoms	−0.054	0.019	0.000	(−0.088, −0.024)
Indirect effect of depressive symptoms	−0.486	0.041	0.000	(−0.558, −0.422)

## Discussion

Suicide has been recognized as a public health priority, particularly during major life events. In the current study, university students considered “drug addict,” “infected with HIV,” and “incurable illness” as the three events that would most likely lead them to consider suicide, indicating that health was the most significant point in suicide acceptability. Furthermore, for suicide prevention, we found that social support negatively predicted suicide acceptability of university students in 25 suicide-related predicaments; moreover, different types of social support had different effect sizes and pathways on suicide risk.

As for suicide acceptability of university students, subjective support and support utilization played the main protective role, while the association between objective support and suicide acceptability turned out to be small. Adolescence and young adulthood are stressful for students and involve identity formation in self-concept, social roles, and interpersonal relationships. According to the interpersonal theory of suicide, a close network and social support contribute to the mental health of adolescents and young adults (18). With a possible explanation for social support's buffer function in previous studies, suicide risk had been decreasing when individuals received more social support from their surroundings (40). All types of social support were associated with increased mental health and decreased suicide acceptability. Perception of spiritual and material support from families, friends, and others, could provide power for university students to deal with life stresses and overcome life dilemma (41). Note that different contexts may not allow students to fully draw on the benefits of social support. Our findings indicated that social support could play a protective role against suicide acceptability among Chinese university students only when it was perceived. This suggested an urgent need to cultivate adolescents' ability to perceive social support and their gratitude for providers of social support.

Furthermore, the results of this study showed that anxiety and depressive symptoms of university students played mediation roles in the associations between all types of social support and suicide acceptability. Particularly, subjective support and support utilization not only had a significant direct negative effect on suicide acceptability but also had indirect effects *via* reducing anxiety and depressive symptoms; however, the protective impact of objective support on suicide acceptability totally worked through decreasing anxiety and depressive symptoms. Chinese culture is highly collectivist (42). Chinese university students are more likely to seek social support from members of the in-group, such as families, peers, classmates, and teachers. Social support, one of the most important protective factors for adolescence to adjust to university, contributes to fewer negative feelings, such as loneliness (43), anxiety, and depressive symptoms (44). In China setting, university students had more opportunities to get good social support and then recovery from negative life events. Objective support, such as financial assistance, was negatively associated with suicide risk by improving individual mental health. Subjective support had impacts on both mental health and suicide risk in this study, suggesting that university students needed essential subjective care and help from families, peers, and society, not only objective support. Support utilization was essential in the reduction of suicide acceptability. More perceived social support was related to increased personal growth and decreased mental health problems (45). After reducing psychological distress, particularly anxiety and depressive symptoms, university students live with hope, motivation, and confidence for life, and then suffer from lower suicide risk (46). Moreover, the mediation effects of depressive symptoms turned out to have stronger power than anxiety in the protective impact of all types of social support on suicide risk in this study. When establishing a social support network, more attention should be paid to reducing the level of depression among university students. Improvement of mental health benefited from providing more individualized care and support for university students.

Overall, the current study indicated that more attention should be given to university students who encountered negative life events: “drug addict,” “infected with HIV,” and “incurable illness.” Social support played an important protective role against suicide risk, as well as in mental health. In this study, mental health served as a nice explanation of the protective mechanisms of social support on suicide acceptability. From the mental health perspective, families, friends, classmates, teachers, and communities should work together to establish and maintain a better social support system for university students, if necessary, help them seek professional mental health services.

### Limitations

There were some limitations. First, since the study was cross-sectional, we could not draw the conclusion that the associations between factors were truly causative. Second, we assessed the suicide acceptability level with the 25-item scale of suicide acceptability, which was created in the Chinese university setting. We are not sure whether our results generalize to other populations. Third, depressive symptoms were assessed with the Beck Depression Inventory, which was a scale focused on the psychological symptoms of depression. Future studies should use a clinician-rated scale such as the Hamilton Depression Rating Scale, which is more focused on both physical and psychological symptoms of depression (47). Moreover, given the self-report instruments, there might be response bias. Improvement of assessment instruments is necessary for future studies.

## Conclusions

Among Chinese university students, suicide acceptability was elevated when there was a health scare. “Drug addict,” “infected with HIV,” and “incurable illness” were the top three events for accepting suicide. Social support could effectively protect adolescents and young adults away from suicide risk. Specifically, social support not only had a direct negative effect on suicide acceptability but also reduced the suicide risk *via* decreasing anxiety and depressive symptoms.

## Data Availability Statement

The raw data supporting the conclusions of this article will be made available by the authors, without undue reservation.

## Ethics Statement

The studies involving human participants were reviewed and approved by the Ethics Committee of Harbin Medical University. Written informed consent to participate in this study was provided by the participants' legal guardian/next of kin.

## Author Contributions

HC: conceptualization, methodology, software, formal analysis, validation, visualization, writing—original draft, and writing—review and editing. YY: conceptualization, data curation, project administration, resources, supervision, and writing—review and editing. JZ, WW, XQ, XY, ZQ, XS, and EZ: investigation. All authors: contributed to the article and approved the submitted version.

## Conflict of Interest

The authors declare that the research was conducted in the absence of any commercial or financial relationships that could be construed as a potential conflict of interest.
